# Examining the Impact of Digital Detox Interventions on Anxiety and Depression Levels Among Young Adults

**DOI:** 10.7759/cureus.75625

**Published:** 2024-12-12

**Authors:** Turki M Alanzi, Wejdan Arif, Reem Aqeeli, Aasal Alnafisi, Tarteel Qumosani, Afrah Alreshidi, Swmayah Alhawsawi, Rabab Alnakli, Abdulelah Alotaibi, Munirah AlOthman, Moruj Khamisi, Nouf Alanzi

**Affiliations:** 1 Health Information Management and Technology Department, College of Public Health, Imam Abdulrahman Bin Faisal University, Dammam, SAU; 2 Department of Radiological Sciences, King Saud University, Riyadh, SAU; 3 Pharmacy, Armed Forces Hospital, Wadi Al Dawasir, SAU; 4 College of Medicine, King Saud Bin Abdulaziz University for Health Sciences, Jeddah, SAU; 5 Department of Public Health, College of Public Health, University of Hail, Hail, SAU; 6 Pharmaceutical Services, Armed Forces Hospital, Wadi Al Dawasir, SAU; 7 College of Pharmacy, Qassim University, Buraidah, SAU; 8 Pharmaceutical Science, Prince Sattam Bin Abdulaziz University, Al-Kharj, SAU; 9 College of Pharmacy, Jazan University, Jazan, SAU; 10 Clinical Laboratory Sciences, Jouf University, Jouf, SAU

**Keywords:** anxiety, depression, digital detox, intervention, mental health, young adults

## Abstract

Background: With increasing reliance on digital devices, concerns about their impact on mental health have grown, particularly among young adults.

Aim: This study aims to evaluate the impact of a digital detox intervention on reducing anxiety and depression among young adults across diverse demographic backgrounds.

Methods: A pre-test, followed by a digital detox intervention, and a post-test using an online survey was carried out. The sample comprised 467 participants (51.6% males, 48.4% females) aged 18-30 years, with varied employment statuses. Participants' anxiety and depression levels were assessed before and after a two-week-long digital detox intervention, which encouraged reduced digital device usage. Data were analyzed using ANOVA to examine differences in mental health outcomes across demographic groups.

Results: Findings indicated statistically significant reductions in anxiety and depression scores post-intervention, with p-values < .0001 for all comparisons. Anxiety scores for males dropped from a mean of 12.50 (variance = 11.08) to 6.58 (6.20), while females showed a decrease from 14.74 (8.92) to 8.29 (6.45). Similarly, depression scores significantly declined from moderate to mild levels across gender, age, and occupational categories. For example, full-time employees’ depression scores decreased from 13.47 (10.44) to 6.80 (5.71), and unemployed participants' scores dropped from 13.95 (11.61) to 7.13 (6.43). Participants reported varied impacts of anxiety and depression on daily responsibilities, with nearly half finding these tasks “Somewhat difficult.”

Conclusion: Digital detox can significantly reduce anxiety and depression symptoms across young adult demographics, underscoring its potential as a non-clinical intervention for mental health. These findings support the inclusion of structured digital disengagement periods in mental health management strategies in educational and occupational settings.

## Introduction

In today’s digitally connected world, smartphones, computers, and other devices have become indispensable parts of everyday life. While the benefits of constant connectivity are undeniable, such as easier communication, access to information, and the ability to multitask [1,2], the downside of prolonged and uninterrupted use of digital devices has become an increasing concern, especially among young adults [3-5]. Recent studies have shown that excessive screen time and continuous interaction with digital technology can have profound effects on mental health, leading to heightened levels of anxiety, depression, and other psychological issues [6-9]. This phenomenon has given rise to the concept of “digital detox,” which involves taking intentional breaks from the use of digital devices to improve mental well-being [10]. The growing awareness of the adverse effects of the overuse of digital technology has sparked interest in understanding how digital detox interventions can influence mental health, particularly in terms of reducing anxiety and depression.

The importance of exploring the relationship between digital detox practices and mental health, specifically anxiety and depression, among young adults cannot be overstated. This demographic is particularly susceptible to the negative impacts of excessive digital engagement [11]. Young adults are often the heaviest users of smartphones and other digital devices, spending significant amounts of time on social media, gaming, and online communication [12,13]. Studies have suggested that this constant connectivity may foster feelings of isolation, anxiety, and even depression due to phenomena such as social comparison, cyberbullying, and sleep disruption [14,15]. As such, young adults represent a critical group for understanding the impact of digital detox interventions on mental health. By examining the effect of these interventions, this study aims to contribute valuable insights into potential strategies for improving psychological well-being in an era of digital overload.

This study is particularly significant within the context of Saudi Arabia, where the prevalence of digital device usage is exceptionally high, with 36.84 million internet users, 99% internet penetration, and a total of 49.89 million cellular mobile connections (134.1% of the total population) in January 2024 [16]. Saudi Arabia has one of the world’s largest and fastest-growing populations of smartphone users, with an increase of 17.95 million users in 2019 to 24.8 million users in 2024 [17]. The Kingdom’s Vision 2030 initiative, aimed at transforming the country into a digital economy, has further accelerated the adoption of digital technologies across all aspects of life, including education, work, and social interactions. Young adults in Saudi Arabia are at the forefront of this digital revolution, spending increasing amounts of time online for both educational purposes and leisure activities, with an average screen time of seven hours and 45 minutes per day [18]. While this technological advancement has brought about numerous benefits, it has also raised concerns about the potential negative mental health outcomes, such as anxiety, depression, and stress associated with excessive screen time, and continuous digital engagement [19,20].

In recent years, mental health has become a critical issue in Saudi Arabia, especially as the country undergoes rapid social and economic changes [21]. Young adults are experiencing new pressures, including academic stress, career uncertainties, and social expectations, all of which can exacerbate feelings of anxiety and depression. Furthermore, the stigma surrounding mental health in Saudi Arabia often prevents individuals from seeking help, meaning that many young adults may suffer in silence [22-24]. The combination of these societal pressures and increased digital consumption makes Saudi Arabia an ideal setting for examining the effectiveness of digital detox interventions as a potential tool for alleviating anxiety and depression among young adults.

The cultural context of Saudi Arabia also adds a unique dimension to this study. In a conservative society, face-to-face interactions have traditionally been more limited, and much of the social interaction among young adults occurs online [25,26]. This reliance on digital platforms for social engagement can contribute to feelings of isolation, particularly if online interactions do not provide the emotional support or connections that individuals need. Moreover, the pandemic has further intensified digital engagement, with many young adults shifting to online learning and remote work, further blurring the boundaries between personal and digital lives [27].

By conducting this study in Saudi Arabia, this study explores whether digital detox intervention can provide an effective strategy for reducing anxiety and depression levels among young adults in a society where digital usage is so deeply ingrained. This research is particularly timely given the growing awareness of mental health issues in the region and the government’s efforts to address these challenges as part of its broader healthcare reforms. By shedding light on the potential benefits of digital detoxing, this study could inform policy recommendations and intervention strategies aimed at improving the mental well-being of young adults in Saudi Arabia. Ultimately, the findings could contribute to a better understanding of how to manage the psychological impact of living in an increasingly digital world, not only in Saudi Arabia but also globally.

This study aims to (1) examine the impact of structured digital detox interventions on reducing anxiety and depression levels among young adults, (2) analyze variations in intervention effectiveness across demographic groups such as age, gender, and employment status, and (3) explore participants’ perceptions and experiences with digital detox practices to identify potential barriers and facilitators for broader implementation.

Literature review

The rapid proliferation of digital technology has significantly transformed the daily lives of young adults, leading to pervasive engagement with social media, mobile devices, and other digital platforms. While these technological advancements offer numerous benefits, such as increased connectivity and access to information, they also present substantial risks to mental health [28]. The growing concern over the negative impact of excessive digital media use has led to the emergence of "digital detox" interventions, which aim to mitigate these risks by encouraging individuals to disconnect from their digital devices for specific periods [29]. This literature review examines existing research on the relationship between digital media use, mental health outcomes, and the efficacy of digital detox interventions in reducing anxiety and depression among young adults.

A substantial body of literature [30-33] has documented the association between excessive digital media use and adverse mental health outcomes, particularly among young adults. For instance, a recent study [34] found that prolonged exposure to social media correlates with increased levels of anxiety, depression, and stress. The negative impact is often attributed to social comparison, cyberbullying, and disrupted sleep patterns, which are exacerbated by the ubiquitous nature of digital devices. Similarly, another study [35] reported a significant rise in depressive symptoms among adolescents and young adults that coincided with the surge in smartphone use and social media engagement.

Moreover, the addictive nature of digital technology has been linked to mental health challenges. Research studies have highlighted that internet addiction, characterized by compulsive use and an inability to control digital consumption, is closely associated with anxiety and depression [36,37]. The constant need for validation and fear of missing out (FOMO) are key factors driving this compulsive behavior, leading to a detrimental cycle of increased digital engagement and deteriorating mental well-being [38].

In response to the growing concerns over digital media's impact on mental health, digital detox interventions have gained popularity as a potential solution. Digital detox refers to a period during which individuals voluntarily refrain from using digital devices, with the goal of reducing stress, improving sleep, and enhancing overall well-being [29]. Several studies have explored the effectiveness of such interventions, though the evidence remains mixed. A recent study observed that there is little evidence for increases in the associations between adolescents’ technology engagement and mental health, except for the use of social media leading to emotional problems [39].

A study has demonstrated that participants who engaged in a one-week digital detox reported significant improvements in mental well-being, including reduced anxiety and depressive symptoms [40]. The study found that the absence of constant digital distractions allowed individuals to engage in more meaningful offline activities, such as face-to-face interactions and physical exercise, which are known to have positive effects on mental health. Similarly, another study [41] found that short-term digital detox interventions led to reduced stress levels and improved mood, particularly among individuals who exhibited high levels of digital dependency. However, both studies were conducted in different geographical regions and focused on specific social media detox only.

## Materials and methods

Study settings and participants

The study was conducted among young adults in Saudi Arabia, aged between 18 and 30 years, who were either university students or early-career professionals, representing a demographic with high digital device usage [40,41]. The age range of 18-30 was selected as it represents a critical developmental stage characterized by significant life transitions, including higher education, early career establishment, and relationship formation. These transitions often amplify digital engagement due to academic, professional, and social networking demands, making this group particularly vulnerable to digital dependency and its associated mental health effects. Additionally, the preferences and digital consumption patterns of individuals in this age range differ markedly from older age groups, who may exhibit different psychological resilience, coping strategies, and levels of digital engagement.

Participants were recruited through social media platforms, university networks, and professional organizations by sharing an invitation link to ensure a diverse sample in terms of background and daily digital usage habits. The study took place over a period of two weeks, during which participants were required to engage in digital detox interventions aimed at reducing their use of digital devices, particularly smartphones, computers, and social media, for specific time intervals (less than 30 minutes each day or in 24 hours) or completely avoiding their use. A pre-test survey was conducted before the intervention, and a post-test survey was conducted after the intervention to measure the anxiety and depression levels. Informed consent was obtained from all participants prior to their inclusion in the study. To be eligible, participants needed to report high levels of daily digital device use, defined as five or more hours per day [40], and they should have experienced some symptoms of anxiety or depression, based on a pre-study screening questionnaire, and reside in Saudi Arabia. Exclusion criteria included individuals with diagnosed mental health disorders that required ongoing medical treatment or counseling, as the study aimed to focus on minimal to severe levels of anxiety and depression, and those who did not follow digital detox practices for a minimum of two weeks. 

Sampling

This study employed a purposive sampling method to recruit participants who met specific inclusion criteria relevant to the research objectives [42]. Young adults aged 18 to 30 years residing in Saudi Arabia were targeted for their high digital device usage and susceptibility to anxiety and depression linked to excessive screen time. Participants were recruited through an online survey distributed via social media platforms, email networks, and community forums to reach a diverse audience from regions including Riyadh, Mecca, Jeddah, and Dammam. These platforms were chosen for their broad accessibility and ability to engage participants across geographic and demographic groups. The sample size was determined using Cochran’s formula for calculating sample sizes in large populations, which is widely used in cross-sectional surveys to ensure statistical reliability and validity [43].

Sample size calculation

Cochran’s formula is given as:​​​​​

n_0_=(Z^2^*p*(1-p))/e^2^

where n_0_: Initial sample size, Z: Z-value for the desired confidence level (1.96 for 95%), p: Estimated proportion of the population exhibiting the attribute of interest (assumed at 50% to maximize variability) and e: Margin of error (5%, or 0.05)

Using this formula, the estimated sample size is calculated to be 383. Given the study's target population size (blood donors in Saudi Arabia), a finite population correction factor was not applied, as the population was considered sufficiently large. To account for potential non-responses and incomplete surveys, the sample size was increased by 20%, leading to a target of approximately 460 participants. This adjusted sample size ensured adequate statistical power to detect significant differences across demographic groups and geographic locations. A total of 479 participants were included in the study, however, 12 participants did not complete post-study survey, leading to a final sample of 467 participants.

Study instruments

The instruments used in this study included self-reported questionnaires to measure anxiety, depression, and digital detox practices among participants. The Generalized Anxiety Disorder scale (GAD-7) was utilized to assess anxiety levels, while the Patient Health Questionnaire (PHQ-9) was employed to measure depressive symptoms. Both scales are widely validated and reliable tools for assessing anxiety and depression in clinical and non-clinical populations, making them appropriate for this study's focus on young adults with mild to moderate mental health concerns [44,45]. To assess participants' digital detox behaviors, a custom-designed Digital Detox Practices Questionnaire (DDPQ) (Appendix) [29,46] was developed based on existing literature on digital detox interventions, which was used in the post-study survey. Arabic versions of PHQ-9 and GAD-7 [47] were also considered. The survey is provided in both Arabic and English languages to ensure accessibility and understandability for the large number of donors. 

The items in both questionnaires were rated on a four-point Likert scale (0: Not at all; 1: Several days; 2: More than half the days; 3: Nearly every day). Anxiety scores are classified into four categories based on severity. Scores ranging from 0 to 4 indicate minimal anxiety, suggesting little to no distress. Individuals scoring 5 to 9 experience mild anxiety, reflecting manageable symptoms that may not significantly impair daily functioning. A score between 10 and 14 corresponds to moderate anxiety, characterized by more noticeable symptoms that could affect productivity and interpersonal interactions. Finally, scores from 15 to 21 signify severe anxiety. Depression scores are categorized to reflect the severity of symptoms. Scores between 1 and 4 indicate minimal depression, suggesting negligible emotional distress. Individuals scoring 5 to 9 experience mild depression, with symptoms that are noticeable but may not significantly disrupt daily life. A score of 10 to 14 denotes moderate depression, characterized by more pronounced symptoms that can interfere with routine activities. Scores ranging from 15 to 19 indicate moderately severe depression, often involving substantial distress and functional impairment. Finally, scores between 20 and 27 reflect severe depression.

Data collection

The data collection process for this study followed a structured approach, beginning with a pre-intervention survey to establish baseline levels of anxiety, depression, and digital device usage behaviors among participants. Before the intervention, participants completed a set of standardized self-reported questionnaires, including the GAD-7 and PHQ-9. These pre-intervention survey measures provided initial data on participants' mental health and their engagement with digital devices, which were used to assess changes in anxiety and depression scores following the intervention.

After the pre-intervention survey, participants underwent a two-week digital detox intervention. During this period, they were instructed to intentionally reduce their use of digital devices, particularly smartphones, computers, and social media. Specific guidelines were provided to help participants limit their digital exposure, such as scheduling daily digital-free periods, avoiding non-essential screen time, and engaging in offline activities. Participants were encouraged to keep a personal log of their digital detox experiences, recording how often and for how long they disconnected from their devices, as well as any challenges or benefits they observed. Regular reminders and support were provided through email or messaging to encourage adherence to the detox plan [10,29,41,46,48]. Following the two-week intervention, participants completed a post-intervention survey that mirrored the pre-intervention survey, using the same set of questionnaires (GAD-7, PHQ-9) and digital practices questionnaire to assess any changes in their anxiety, depression, and digital device usage habits.

Data analysis

The post-test data allowed for a comparison between the pre-and post-intervention scores, enabling the researchers to evaluate the effectiveness of the digital detox intervention in reducing anxiety and depression levels among the participants. To achieve the study's objectives, the data were analyzed using the Statistical Package for the Social Sciences software, version 24.0 (IBM Corp., Armonk, NY). Descriptive statistics, including means and standard deviations, were employed to present the demographic characteristics of the participants. Additionally, a two-sample t-test with unequal variances was conducted to analyze the data further.

Ethical considerations

The study received approval from the Research Ethics Committee at Imam Abdulrahman Bin Faisal University (IRB-2024-03-605). Prior to data collection, informed consent was obtained from all participants. As the survey was conducted online, participants provided electronic consent by selecting a checkbox on the first page of the survey, indicating that they had read and understood the purpose of the study, the voluntary nature of their participation, and the confidentiality of their responses. The consent form included detailed information about the study's objectives, the types of data being collected, the measures in place to ensure anonymity, and the right to withdraw at any point without consequence. Only participants who provided consent were able to proceed with the survey, ensuring that all responses were collected ethically. The study adhered to all relevant ethical standards (The Declaration of Helsinki), with no reported conflicts of interest or external funding sources, thereby upholding research integrity and minimizing bias.

## Results

The demographic data in Table 1 reveals a fairly balanced gender distribution among participants, with 51.6% male (241 individuals) and 48.4% female (226 individuals). The age distribution spans from 18 to 30 years, with the largest group being 27-28 years (22.7%), followed by those aged 18-20 (20.6%) and 24-26 (19.9%). Participants aged 21-23 and 29-30 years constitute 19.1% and 17.8% of the sample, respectively. In terms of occupation, the sample includes a mix of employment statuses, with 22.1% employed part-time, 21.2% employed full-time, 20.6% unemployed, 20.1% students, and 16.1% retired. This diverse distribution across age, gender, and occupation suggests a well-rounded participant base, likely enhancing the generalizability of the study findings.

**Table 1:** Participants demographics

Factors	Variables	N	Relative frequency
Gender	Male	241	51.6%
	Female	226	48.4%
Age (in years)	18-20	96	20.6%
	21-23	89	19.1%
	24-26	93	19.9%
	27-28	106	22.7%
	29-30	83	17.8%
Occupation	Employed full-time	99	21.2%
	Employed part-time	103	22.1%
	Student	94	20.1%
	Unemployed	96	20.6%
	Retired	75	16.1%

Impact of digital detox intervention on anxiety

The ANOVA results in Table 2 indicate statistically significant reductions in anxiety scores among participants following a digital detox intervention, with all comparisons yielding p-values of < .0001. This significance suggests that the intervention effectively reduced anxiety across all demographic groups. Gender-wise, males had a higher reduction in mean anxiety scores, decreasing from 12.50 to 6.58, compared to females, whose scores dropped from 14.74 to 8.29. Age-wise, all groups experienced reductions, with younger participants (18-20 and 21-23 years) showing lower initial scores and smaller variances post-intervention, while older participants (27-30 years) had higher initial scores and variances. By occupation, full-time and part-time employees, students, and retirees all showed marked decreases, with unemployed individuals experiencing the largest initial scores (15.17) and highest post-intervention variance. The consistent reductions across demographic categories underscore the digital detox’s potential efficacy in alleviating anxiety among young adults.

**Table 2:** Differences in the anxiety scores of the participants before and after the digital detox intervention * Statistically significant difference at .05 CI

Factors	Variables	Before intervention			After intervention		p-value
		N	Mean anxiety score	Variance	Mean score	Variance	
Gender	Male	241	12.50	11.08	6.58	6.20	< .0001*
	Female	226	14.74	8.92	8.29	6.45	< .0001*
Age (in years)	18-20	96	12.81	9.73	6.88	5.75	< .0001*
	21-23	89	12.58	11.36	6.71	6.69	< .0001*
	24-26	93	12.86	9.40	6.80	5.29	< .0001*
	27-28	106	14.65	10.53	8.21	7.29	< .0001*
	29-30	83	15.00	10.68	8.45	7.69	< .0001*
Occupation	Employed full-time	99	13.45	11.09	7.29	6.90	< .0001*
	Employed part-time	103	12.47	12.11	6.57	6.46	< .0001*
	Student	94	12.62	10.97	6.55	6.77	< .0001*
	Unemployed	96	15.17	10.04	8.72	7.15	< .0001*
	Retired	75	14.48	5.93	8.11	3.96	< .0001*

Before the intervention, participants across most demographic categories generally scored in the moderate to severe anxiety range, with mean scores around 12.50 to 15.00. This places many participants in moderate anxiety (10-14) and some in severe anxiety (15-21), indicating a baseline of considerable anxiety levels. After the intervention, mean anxiety scores dropped to a mild range (5-9) across all groups, showing that the digital detox had a meaningful impact in reducing anxiety. This pattern holds for different age groups and occupations, with post-intervention scores in the mild range (5-9), reflecting reduced anxiety across all demographics.

**Figure 1:**
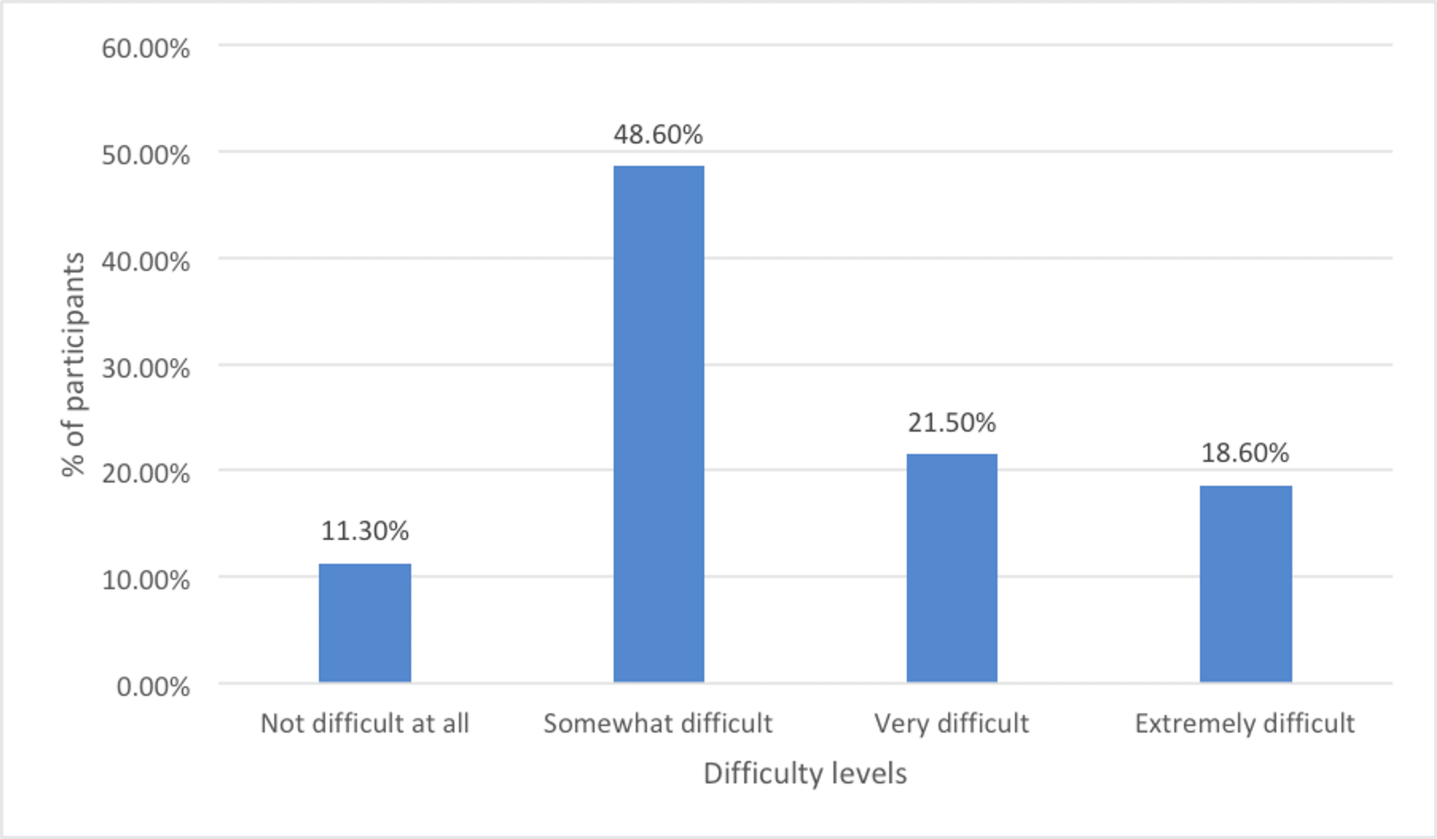
The ability of study participants to handle daily responsibilities Difficulty in doing work, taking care of things at home, or getting along with other people due to anxiety problems.

Figure 1 reveals that anxiety has a varying impact on individuals' ability to handle daily responsibilities, including work, home tasks, and relationships. Only 11.3% of participants reported that these activities were "Not difficult at all" despite their anxiety, suggesting minimal impact for a small group. In contrast, nearly half of the participants (48.6%) found these activities "Somewhat difficult," indicating moderate challenges for the majority. A notable 21.5% of individuals reported their tasks as "Very difficult" to manage, highlighting substantial difficulties, while 18.6% found them "Extremely difficult," showing that almost one-fifth of the participants faced severe impairments due to anxiety. This distribution underscores the wide-ranging impact of anxiety, with many experiencing moderate to severe difficulties in daily functioning.

Impact of digital detox intervention on depression

The ANOVA results in Table 3 reveal significant reductions in depression scores across all demographic groups following the digital detox intervention, with all p-values < .0001. Gender-wise, both males and females initially had mean scores in the moderate depression range (10-14), with males at 13.54 and females at 13.72. After the intervention, their scores dropped to the mild depression range (5-9), with post-intervention means of 6.78 for males and 6.92 for females, indicating substantial improvement.

**Table 3:** Differences in the depression scores of the participants before and after the digital detox intervention * Statistically significant difference at .05 CI

Factors	Variables	Before intervention			After intervention		p-value
		N	Mean depression score	Variance	Mean score	Variance	
Gender	Male	241	13.54	11.20	6.78	6.24	< .0001*
	Female	226	13.72	12.04	6.92	6.90	< .0001*
Age (in years)	18-20	96	13.91	11.14	6.90	6.24	< .0001*
	21-23	89	13.07	11.20	6.46	5.89	< .0001*
	24-26	93	13.80	10.06	6.92	5.74	< .0001*
	27-28	106	14.19	13.16	7.32	6.77	< .0001*
	29-30	83	13.00	11.51	6.54	8.01	< .0001*
Occupation	Employed full-time	99	13.47	10.44	6.80	5.71	< .0001*
	Employed part-time	103	13.73	11.42	6.89	6.57	< .0001*
	Student	94	13.49	13.05	6.71	7.45	< .0001*
	Unemployed	96	13.95	11.61	7.13	6.43	< .0001*
	Retired	75	13.45	11.87	6.69	6.86	< .0001*

Age-wise, all groups also saw reductions from moderate depression levels (mean scores ranging from 13.00 to 14.19) to mild depression levels post-intervention. The 21-23 years age group showed the largest improvement, decreasing from 13.07 to 6.46, while the 27-28 years age group retained the highest post-intervention mean score at 7.32, though still within the mild range.

Occupation-wise, each category experienced significant reductions, with scores moving from moderate to mild depression. For instance, full-time employees’ scores dropped from 13.47 to 6.80, and unemployed participants’ scores went from 13.95 to 7.13. This consistent shift from moderate to mild depression across all demographics highlights the digital detox intervention's effectiveness in reducing depression symptoms for a wide range of participants.

**Figure 2:**
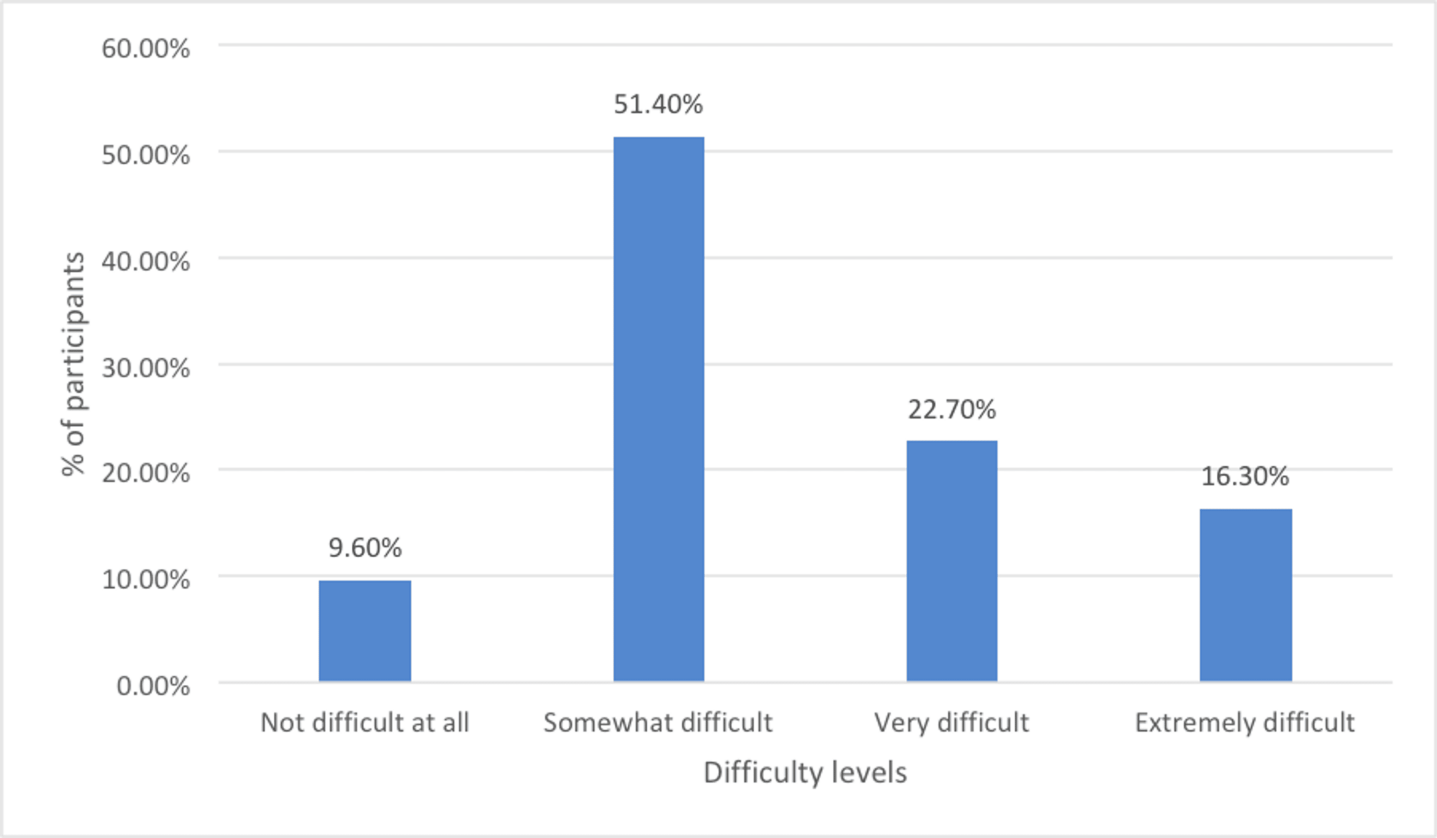
The ability of study participants to handle daily responsibilities after the digital detox intervention Difficulty in doing work, taking care of things at home, or getting along with other people due to depression problems.

Figure 2 represents the distribution of respondents based on the level of difficulty they experience in performing tasks, such as doing work, taking care of things at home, or getting along with other people due to depression-related problems. According to the data, a majority of respondents (51.4%) find these activities "Somewhat difficult," suggesting that while they can manage their responsibilities, depression imposes a noticeable challenge. A smaller portion (22.7%) reports that these tasks are "Very difficult," indicating significant disruption in daily functioning. Additionally, 16.3% of the respondents describe their difficulty as "Extremely difficult," highlighting severe impairment due to depression. Only 9.6% of respondents report "Not difficult at all," meaning depression does not substantially affect their ability to carry out daily tasks. Overall, the data suggests that depression has varying levels of impact on individuals' ability to perform everyday tasks, with the majority facing at least some level of difficulty.

Table 4 reflects participants' digital detox practices and perceptions, with mean scores calculated on a scale from 1 (strongly disagree) to 5 (strongly agree). Participants moderately agree that they regularly take breaks from digital devices (mean score = 3.30) and that they have achieved complete digital detoxing (mean score = 3.31), indicating that while digital detoxing is somewhat practiced, it is not universally adopted. Scheduling specific times to avoid digital devices has a similar mean score of 3.22, suggesting moderate engagement in structured detox practices. However, participants also find it somewhat difficult to go without digital devices for more than a few hours, as indicated by a mean score of 3.35, reflecting the challenge of disconnecting. The highest level of agreement is on the relief participants feel when they take breaks from digital devices (mean score = 3.74), indicating a positive emotional response to digital detoxing. The variance scores suggest there is some variation in participants' engagement with these practices, with the highest variance (1.97) seen in the difficulty of going without devices for extended periods.

**Table 4:** Digital detox practices and perceptions

Digital detox practices and perceptions	Mean score	Variance
I regularly take breaks from using digital devices (e.g., phone, computer, TV).	3.30	1.49
I have achieved digital detoxing (completely disconnecting from digital devices).	3.31	1.53
I schedule specific times during the day where I avoid using digital devices.	3.22	1.50
I find it difficult to go without digital devices for more than a few hours	3.35	1.97
I experience a sense of relief when I take a break from digital devices.	3.74	1.03

## Discussion

The results of this study provide significant insights into the impact of digital detox interventions on anxiety and depression levels among young adults. The key findings indicate a positive association between digital detox and improved mental health, specifically in reducing anxiety and depression. This is consistent with previous studies [30-38], which highlighted the adverse effects of prolonged digital engagement on mental health, and studies [40,41], which highlighted the positive association between digital detox and improved mental health. However, our study not only reaffirms these findings but also adds to the growing body of evidence by emphasizing the potential of structured digital detox interventions in mitigating these negative effects.

A critical observation from our findings and literature review is that the duration and frequency of digital detox sessions play a crucial role in determining the effectiveness of the intervention. Participants who engaged in regular, shorter detox periods reported more substantial mental health improvements than those who opted for longer but infrequent detoxes. This is in contrast to [48], which suggested that more extended detox periods might be necessary for noticeable improvements in well-being. Thus, suggesting that consistency, rather than length, is the key to success. These findings are particularly important in the context of modern lifestyles, where the feasibility of extended detox periods might be limited.

The digital detox intervention led to a statistically significant reduction in anxiety across all demographic groups. Gender-wise, males experienced a greater reduction in anxiety scores than females, aligning with findings in [41], who reported that men might respond more favorably to digital detox due to differences in coping mechanisms and stress reactivity. However, this finding diverges from [49], which suggested that females might benefit more from digital disengagement interventions due to their higher baseline social media usage and susceptibility to online stressors. Future studies could explore these gender differences further, examining whether specific intervention components are more effective for one gender over the other.

Age-wise, the intervention was effective across all age groups, though younger participants (18-23 years) showed a more modest reduction compared to older participants (27-30 years). This pattern supports findings in [50], which noted that older adults, despite lower digital consumption, experience heightened stress from digital interactions and thus benefit more from disengagement. However, many studies [3-5,12,13,18-20] found younger adults more susceptible to anxiety from social media overuse, suggesting that digital detox interventions might require age-targeted strategies to maximize effectiveness for younger populations.

In terms of occupational status, unemployed participants had the highest baseline anxiety levels and variance post-intervention, underscoring the link between employment status and mental health. This finding indicates that unemployment is strongly associated with elevated anxiety and depression due to financial insecurity and social isolation. Moreover, employed individuals, especially part-time workers, showed significant anxiety reductions post-detox, which suggests that digital disengagement could serve as a helpful mental health intervention within work environments.

The study also revealed substantial reductions in depression scores across demographics, moving from moderate to mild levels post-intervention. Similar to anxiety results, females exhibited slightly higher post-intervention depression scores than males, which highlights gender-specific differences in stress processing, with females tending to report higher depressive symptoms even post-intervention. Occupationally, unemployed individuals had the highest initial depression levels, which may reflect a strong correlation between unemployment and depression severity, emphasizing the need for accessible mental health interventions for this demographic.

Strengths, limitations and future research recommendations

This study boasts several strengths, including a diverse participant pool of 467 individuals across various age, gender, and occupational demographics, ensuring broad generalizability. The robust pre-and-post-intervention design with standardized measures like GAD-7 and PHQ-9 adds validity. Additionally, its focus on a culturally relevant context (Saudi Arabia) enriches understanding of digital detox effects within high-tech societies.

While this study provides insights into the benefits of digital detox on mental health, several limitations exist. The study relied on self-reported measures, which may be subject to social desirability bias, where participants underreport symptoms of anxiety or depression. Additionally, the cross-sectional survey design does not permit causal inferences regarding the long-term benefits of digital detox. Future research could address these limitations by using objective mental health assessments and a longitudinal design to assess sustained effects. Moreover, investigating tailored interventions based on gender, age, and occupational status could optimize digital detox strategies for diverse populations, especially considering the nuanced responses observed here.

Theoretical and practical implications

The findings of this study extend the theoretical understanding of digital detox interventions within mental health frameworks, supporting the notion that consistent reduction of digital exposure can alleviate symptoms of anxiety and depression across diverse demographic groups. This aligns with theories of digital overstimulation, which propose that constant connectivity contributes to cognitive and emotional strain. The significant reductions in anxiety and depression scores reinforce the application of these theories, suggesting that digital disengagement serves as a moderating factor that may help buffer the mental health impacts associated with digital overuse. Practically, these results have several implications for workplace and educational settings, where structured digital detox programs could be integrated to improve well-being. For instance, organizations could implement regular "device-free" periods or offer workshops on managing digital consumption. Furthermore, these interventions could be customized to address gender and age differences observed in digital engagement, enhancing their effectiveness. Tailoring digital detox programs based on occupational needs, such as targeted support for unemployed individuals who experience heightened anxiety and depression, could also promote mental health equity and provide accessible, non-clinical mental health support. Overall, the study underscores the value of digital detox as a proactive approach to mental health management in a digitally saturated environment.

## Conclusions

This study highlights the effectiveness of structured digital detox interventions in reducing anxiety and depression levels among young adults, underscoring their potential as a non-clinical tool for mental health improvement. By analyzing demographic variations, the study found that while the intervention benefits all participants, its impact differs across age, gender, and employment status, emphasizing the need for demographic-specific strategies. Additionally, the exploration of participants' perceptions revealed both challenges and benefits associated with digital detox practices, such as overcoming digital dependency and recognizing the emotional relief provided by reduced screen time. However, the study has limitations that warrant consideration. The reliance on self-reported measures may introduce bias, as participants might underreport or overreport symptoms due to social desirability or recall inaccuracies. Additionally, the two-week intervention period does not allow for the evaluation of long-term effects or sustainability of mental health improvements. Lastly, the cultural context of Saudi Arabia, while offering valuable insights, may limit the generalizability of the findings to other regions with different digital consumption patterns and societal norms. Future studies should address these limitations by incorporating objective measures, longer follow-up periods, and cross-cultural comparisons to refine digital detox strategies and assess their broader applicability.

## Appendices

Digital detox practices questionnaire

Please rate the following statements about your experiences and attitudes towards digital detax practices on a scale of 1 to 5 (1: Strongly disagree; 2: Disagree; 3: Neutral; 4: Agree; 5: Strongly agree)

I regularly take breaks from using digital devices (e.g., phone, computer, TV).

I have achieved digital detoxing (completely disconnecting from digital devices).

I schedule specific times during the day where I avoid using digital devices.

I find it difficult to go without digital devices for more than a few hours

I experience a sense of relief when I take a break from digital devices.
